# Stabilized fabrication of anatase-TiO_2_/FeS_2_ (pyrite) semiconductor composite nanocrystals for enhanced solar light-mediated photocatalytic degradation of methylene blue

**DOI:** 10.1039/c8ra02077a

**Published:** 2018-03-27

**Authors:** Jamshaid Rashid, Sahar Saleem, Saif Ullah Awan, A. Iqbal, Rajeev Kumar, M. A. Barakat, Muhammad Arshad, Muhammad Zaheer, Mohsin Rafique, M. Awad

**Affiliations:** Catalysis for Environment and Energy Laboratory, Department of Environmental Science, Faculty of Biological Sciences, Quaid-i-Azam University Islamabad 45320 Pakistan jrashid@qau.edu.pk jamshaidrashid@gmail.com; Department of Electrical Engineering, NUST College of Electrical and Mechanical Engineering, National University of Science and Technology (NUST) Islamabad Pakistan; Institute of Environmental Sciences and Engineering, School of Civil and Environmental Engineering, National University of Sciences and Technology Sector H-12 Islamabad 44000 Pakistan; Department of Environmental Sciences, Faculty of Meteorology, Environment and Arid Land Agriculture, King Abdulaziz University Jeddah 21589 Saudi Arabia; Central Metallurgical R&D Institute Helwan 11421 Cairo Egypt; National Centre for Physics, Nano-Science and Technology Department, Quaid-i-Azam University Islamabad Pakistan; Department of Chemistry, SBA School of Science and Engineering, Lahore University of Management Sciences (LUMS) Lahore 54792 Pakistan; Center for Micro and Nano Devices (CMND), COMSATS Institute of Information Technology Islamabad 45550 Pakistan; UNEP-TONGJI Institute of Environment for Sustainable Development, College of Environmental Sciences and Engineering, State Key Laboratory of Pollution Control and Resource Reuse, Tongji University Shanghai China 200092

## Abstract

A novel visible light active TiO_2_/FeS_2_ semiconductor photocatalyst was synthesized by a simple wet chemical process. X-ray diffraction (XRD) was used to analyze the anatase TiO_2_ and pyrite structures in FeS_2_/TiO_2_ nanocrystals. Scanning electron microscopy (SEM) confirmed the spherical morphology of composite nanocrystals. X-ray photoelectron spectroscopy (XPS) identified the Fe^2+^, S^1−^, Ti^4+^, and O^2−^ oxidation states of relevant species. Energy dispersive X-ray (EDX) analysis was performed for compositional analysis. The measured band gap of the TiO_2_/FeS_2_ nanocomposite system was 2.67 eV, which is smaller than un-doped TiO_2_ (3.10 eV) and larger than FeS_2_ (1.94 eV). The photocatalytic activity of TiO_2_/FeS_2_ was significantly higher than pure FeS_2_ for degrading methylene blue (MB) under solar light irradiation due to the increase in visible light absorption, reduction in band gap energy, and better election–hole pair separation. The photocatalytic degradation of MB was investigated under the influence of solution pH, dye concentrations, and varied catalyst dosage. The optimum degradation (100%) of MB was observed in 180 min and the photocatalysis of MB reduced as the dye concentrations in the solution increased from 15 to 75 mg L^−1^. These results prove that the TiO_2_/FeS_2_ nanocomposite has the stability, recycling, and adaptability for its practical application as a visible light photocatalyst for wastewater treatment. TiO_2_/FeS_2_ showed increased degradation of the organic pollutant; which is confirmed by the increased rate of chemical reaction following pseudo first-order reaction kinetics with the highest rate constant value of 0.0408 m^−1^ having highest *R*^2^ value of 0.9981.

## Abstract 2

A novel visible light active TiO_2_/FeS_2_ semiconductor photocatalyst was synthesized by a simple wet chemical process.

## Graphical abstract

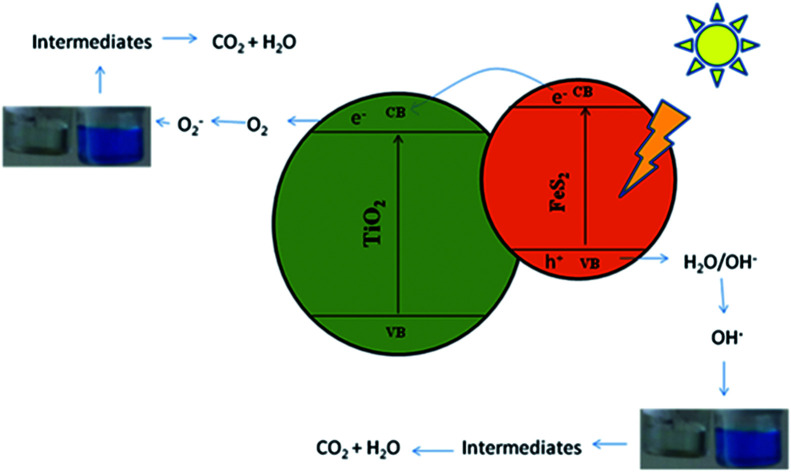

## 1. Introduction

Photocatalytic treatments have been extensively recommended for environmental remediation under benign conditions.^1^ In the presence of a photocatalyst and a beam source of suitable energy, this process can mineralize natural contaminants to nontoxic items such as H_2_O and CO_2_.^2^ Although different physiochemical methods such as adsorption ultrafiltration, reverse osmosis, and biodegradation have been used to remove dyes, all these methods have certain shortcomings. For instance, chemical methods are expensive, require a large dosage of chemicals, and produce hazardous sludge.^3^ Physical methods, on the other hand, transfer pollutants from a liquid phase to solid waste. Biological degradation of dyes has been found to be ineffective and inefficient as they produce aromatic amines. Photocatalytic degradation has been frequently used in wastewater treatment due to its high photoactivity, non-toxicity, low cost, photochemical stability, and unusual capability to degrade pollutants.^4^ Among the semiconductor photocatalysts, titanium dioxide (TiO_2_) nanocrystals are a highly efficient photocatalyst and have been the leading conventional substance for degrading natural impurities.^5,6^ Augmenting to its non-toxicity, abundance, and moderately little cost, TiO_2_ also demonstrates excellent photocatalytic activity in numerous degradation responses. To develop the consumption of solar energy, many investigative attempts have focused on exploring the photocatalytic hydrogen generating potential of TiO_2_ in the visible spectrum (*λ* > 400 nm) which accounts for ∼42% of solar power.^7^

Despite the fact that the photocatalytic properties of TiO_2_ nanocrystals are predominantly controlled by particle dimension, poorer photocatalytic degradation rates are experienced on the exterior surface of TiO_2_ nanoparticles due to their extensive band gap and rapid recombination rate of photogenerated electron–hole pairs.^8^ Consequently, the alteration of TiO_2_ to decrease such recombination remains as a serious assignment.^9^ A significant contribution in this regard can be made by using an environmentally benign and sustainable material as a modifier for TiO_2_ as one of the preeminent modifiers. Employing a co-catalyst has been a predictable way to advance the photocatalytic performances of semiconductor photocatalysts because it enhances charge separation and minimizes photo-corrosion of the semiconductor photocatalyst.^10^ Metal doping,^11^ non-metal doping,^12^ and composites with other semiconductors^13^ also are a few of the potential approaches used to improve the capability of TiO_2_ based materials to utilize solar energy. In recent years, much research has been focused on TiO_2_ composites with chalcogenides because their narrow bandgaps allow absorption of longer wavelengths than pure TiO_2_.^14,15^ Research on metal sulfide photocatalysts has shown growth from the analytical to the application phase, and their short band gaps permit absorption at longer wavelengths than TiO_2_.^10^ Usually, CdS, ZnS, FeS_2_, and MoS_2_ are the most studied sulfide photocatalysts due to their band gap energy, which matches well with the solar spectrum.^16,17^ Among them FeS_2_, also known as pyrite, displays interesting electronic and optical properties as it is an additional constructive applicant for the photosensitization of materials in addition to its environmental compatibilities and high stability toward photo-corrosion as well as high absorption in the visible section of the solar spectrum. In our study, one of the possible co-catalyst modifiers was iron(ii) sulfide (FeS_2_), which is nontoxic, stable, inexpensive, and found in abundance in the world. It can efficiently adsorb and photocatalytically degrade organic dyes^18^ due to its surface chemical properties, high optical absorption coefficient (6 × 10^5^ cm^−1^),^19^ a high capacity (exceeding 890 mA h g^−1^), and suitable band gap *i.e.*, 1.00 ± 0.15 eV^20^ compared to other sulphides. Due to these interesting properties, FeS_2_ has been investigated for applications in photovoltaic devices^21^ and lithium-ion batteries.^22^ FeS_2_ has been prepared by a variety of techniques, including hydrothermal, solvothermal, metal organic chemical vapor deposition, and sulfurization of iron or iron oxide films as reported earlier.^23^

The benefits of integrating two dissimilar metal oxides/sulfide not only progresses photocatalytic activity but also exhibits different physical and chemical properties when evaluated in comparison to constituent metal oxides.^24^ The formation of composites of nanocrystals is a widely explored idea in order to obtain a hybrid which could simultaneously combine the properties of TiO_2_ as a fascinating semiconducting material with the features of single nano-sized metal sulfide particles. The addition of FeS_2_ to TiO_2_ produces a red shift in the absorption edge resulting in a band gap of 2.94–2.84 eV for the composite.^25^ It has been reported^26^ that photocatalytic degradation of MB in the presence of TiO_2_/MoS_2_ composite was increased up to 65% compared to pure TiO_2_ (15%). Similarly,^27^ the spindle-like TiO_2_/CdS composite had a 3.5 times higher photocatalytic efficiency compared to pure CdS. Lee and co-workers^25^ synthesized a FeS_2_/TiO_2_ composite using a solvothermal method; which exhibited 5 times higher hydrogen production compared to pure TiO_2_. Recently, a FeS_2_/TiO_2_ photo-anode was investigated^28^ which exhibited enhanced photo-response from visible light to an extended near IR range (400–900 nm). Few reports of the composites TiO_2_/FeS_2_ quantum dots,^29^ nanotubes,^30^ core shell,^25^ thin films,^31^ and spheres^32^ are reported earlier for applications with solar cells. In this report, we focused on the nanocrystals of composites of the TiO_2_/FeS_2_ system for solar photocatalysis. The present research is focused on developing a photocatalytic system that can effectively utilize sunlight or visible light to degrade commonly used MB dye (a known environmental contaminant). To the best of our information, no study has been conducted to determine the efficiency of composite (TiO_2_/FeS_2_) as a photocatalyst for treatment of MB. We believe the proposed novel FeS_2_/TiO_2_ nanocrystals composite has the prospective to tackle the low efficiency problems of photoelectrochemical (PEC) water splitting in visible and infrared light regions, and therefore can build a noteworthy contribution in the field of energy exchange.

## 2. Materials and methods

### 2.1 Materials

FeSO_4_·7H_2_O and Na_2_S_2_O_3_ were supplied by BDH chemical Ltd and Sigma-Aldrich (USA); TiCl_4_ was purchased from Sigma-Aldrich (USA); Methylene Blue was supplied by BDH chemical Ltd (USA) and standard grade was utilized as the test pollutant. Deionized water was used for solution preparations. All other chemicals were also analytical grade.

### 2.2 Synthesis of TiO_2_/FeS_2_ nanocrystals

In a typical procedure, 3.58 g of FeSO_4_ and 8.5 g of Na_2_S_2_O_3_ were added to 175 mL ethanol and sonicated for 1 h. Then, 2 mL of TiCl_4_ was added to the solution and refluxed at 156 °C for 5 h. A brownish material was filtered and thoroughly washed with deionized water followed by acetone and dried at 80 °C for 12 h. FeS_2_ nanoparticles were synthesized using the same procedure in the absence of TiCl_4_.

### 2.3 Characterization

The crystalline structures of as-prepared composites were characterized using a D8 Bruker X-ray diffractometer by scanning 2*θ* angle ranging from 20 to 80° at a scanning rate of 0.50° per minute using Cu Kα 1.5418 radiation. A Hitachi S-4800 microscope at an operating voltage of 15 kV was used to obtain scanning electron microscope (SEM) images to determine size and shape of the composite. The sample was coated with platinum and copper for effective imaging before being charged. Energy dispersive X-ray (EDAX) was used to determine the elemental composition of particles (EDX, Oxford Instruments, INCA x-sight) with 10 keV electron beam energy. X-ray photoelectron spectroscopy (XPS) measurements were performed for chemical analysis of the TiO_2_/FeS_2_ nanocomposite. XPS data acquisition was performed in ultra-high vacuum conditions using a standard omicron system equipped with monochromatic Al Kα 1486.7 eV X-ray source. The source was operated at 15 keV at constant analyzer energy (CAE) of 100 eV for survey scans and 20 eV for detailed scans. Data acquisition was performed with Matrix software and data analysis was performed with Igor Pro along with XPS fit procedures. Curve fitting of spectra was achieved using Gaussian–Lorentzia line shape after performing Shrilly background corrections. The C 1s binding energy at 284.8 eV was used for calibration. DRS data were recorded using a UV-Vis spectrophotometer (Shimadzu UV-2550) using standard 1 cm quartz cuvettes. A GC (QP2010 ultra, Shimadzu) having a DB-5ms capillary column (30.00 m × 0.25 mm, 0.25 μm film thickness) was coupled to a QP2010 ultra Shimadzu mass selective detector which was used to detect the degradation products. The initial oven temperature (60 °C for 10 min initially) was raised at a rate of 10 °C min^−1^, while injection temperature was 250 °C, and the injection mode was split. Helium was used as a carrier gas. To prepare samples for GC-MS injection, a MB dye solution containing TiO_2_/FeS_2_ composite was filtered, organic compounds were extracted using 5 mL dichloromethane, and analyzed by GC-MS.

### 2.4 Photocatalytic experiments

The photocatalytic activity of the synthesized FeS_2_/TiO_2_ nanocomposite was evaluated under visible and direct sunlight conditions. A Luzchem photochemical reactor (LZC4) was used for visible light investigations with irradiation of 104 W Slovenia cool white lamps (*λ* = 390–700 nm) and spectral irradiance of 17.45 mW cm^−2^ monitored by a Luzchem power monitor at a distance of 12 cm from the light source. Photocatalytic experiments were performed with 250 mL of MB solution at normal solution pH of 5.5 in a Pyrex® glass reactor under continuous stirring and aeration conditions. In typical experiments, duplicate solutions containing 25 mg L^−1^ of MB and 1 g L^−1^ TiO_2_/FeS_2_ nanocomposite (except in case of photolytic studies) were taken and one of the identical solutions was exposed to direct sunlight and the other to visible light. To establish adsorption–desorption equilibrium of MB over the surface of TiO_2_/FeS_2_ nanoparticles, all experiments were carried out for an initial 30 min under dark conditions followed by irradiation with visible/sunlight for 180 min. Aliquots of 3 mL samples were taken at 30 min intervals and filtered through 0.22 μm syringe filters to remove catalyst particles. The residual concentrations of MB in supernatant solutions were determined using a UV-visible spectrophotometer (Hitachi U3000) at 665 nm. To assess the impact of dye concentration on photocatalytic efficiency of TiO_2_/FeS_2_, experiments were performed at initial dye concentrations ranging from 15–75 mg L^−1^. After determination of the optimum dye concentration, additional parameters, including the solution pH and catalyst dose were determined. Comparative studies for direct sunlight photocatalytic degradation of MB were carried out during the months of May and June with average sunlight illumination intensity of 10 000–12 000 lx. The remaining procedure was the same as for visible light studies.

## 3. Results and analysis

### 3.1 X-ray diffraction

**Fig. 1:**
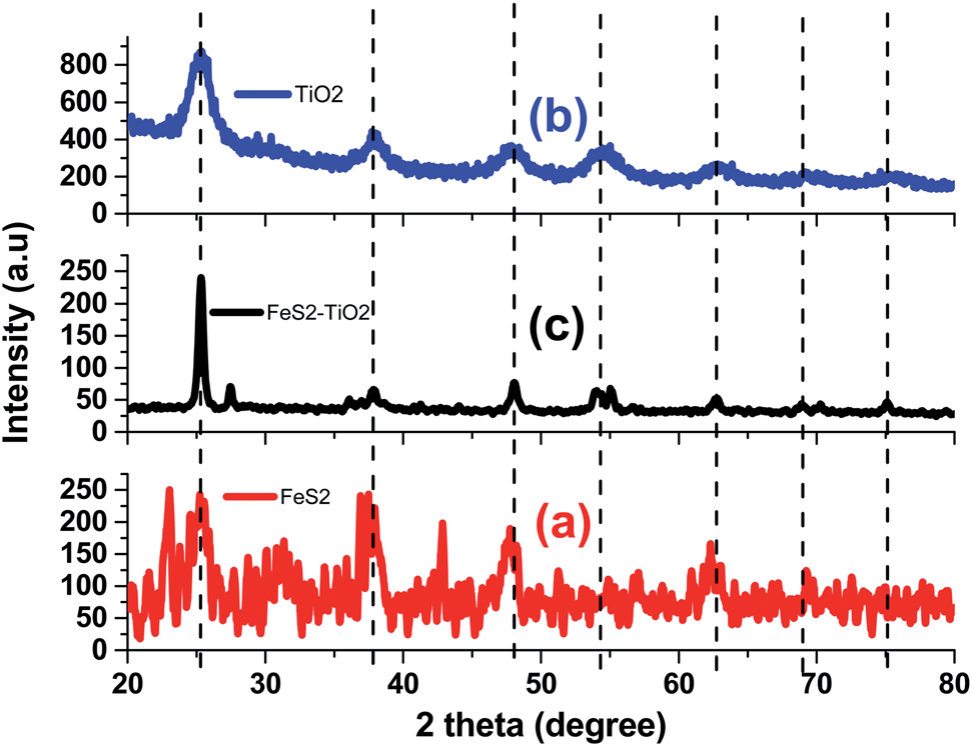
Bragg-diffraction spectrum of (a) FeS_2_, (b) TiO_2_, and (c) TiO_2_/FeS_2_ composite.

Fig. 1(a–c) shows the Bragg diffraction spectrum of polycrystalline FeS_2_, TiO_2_, and composite as synthesized samples, respectively. The diffracted peaks for FeS_2_ (Fig. 1(a)) reflect the single homogeneous phase of FeS_2_ corresponding to cubic phase (JCPDS 00-042-1340) and exhibiting pyrite crystal structure. No extra peaks related to sulfur or any impurity phase such as marcasite, pyrrhotite, or troilite were obtained. Lattice parameters for the cubic structure were measured as: *a* = *b* = *c* = 5.418 Å. The presence of broad peaks in the XRD pattern confirmed the nano-crystalline nature. The average crystallite sizes of pyrite nanocrystals using the Debye–Scherer equation were estimated to be about 15 nm. Fig. 1(b) demonstrated good crystalline single phase anatase-TiO_2_ structures with prominent phase stability along (101) axis. The lattice parameters of anatase TiO_2_ nanocrystals are *a* = 3.7835 Å and *c* = 9.2371 Å. The crystallite size of the anatase TiO_2_ sample is 12 nm.

XRD patterns (Fig. 1(c)) for the TiO_2_/FeS_2_ composite showed mixed pyrite and anatase phases of constituent nanocrystals. The composite sample has a dominant peak which represents a (110) peak of TiO_2_ (anatase) phase. The anatase phase was stable in the presence of FeS_2_ nanocrystals. In the XRD pattern of the composite system, peaks corresponding to a rutile phase were not observed. The pyrite FeS_2_ phase could be clearly observed in the XRD patterns of the composite. The composite sample has another significant peak at ∼48° which corresponds to the (121) peak of the FeS_2_ phase. The intensity of the peak at 2*θ* = 25.85 degree of anatase TiO_2_ NPs (JCPDS card no. #84-1286) decreased as the FeS_2_ content was added into the composite systems. The composite sample has another significant peak at ∼48° corresponding to the (121) peak of the FeS_2_ phase.

### 3.2 Microstructural measurements

**Fig. 2:**
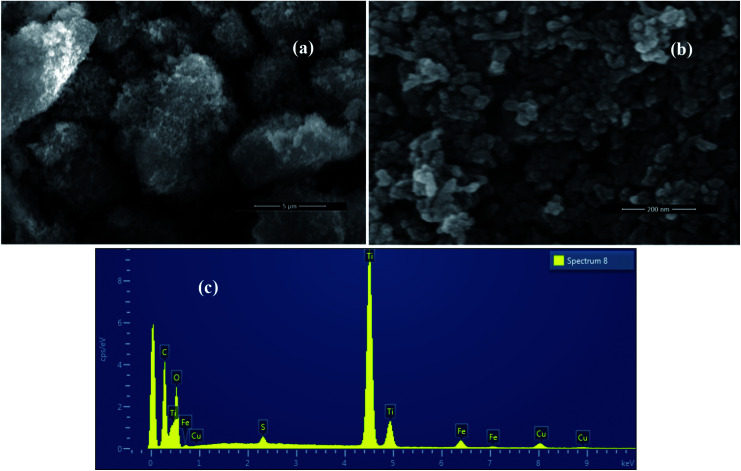
SEM images of synthesized (a) TiO_2_/FeS_2_, (b) FeS_2_ nanoparticles, and (c) EDAX of TiO_2_/FeS_2_.

**Fig. 3:**
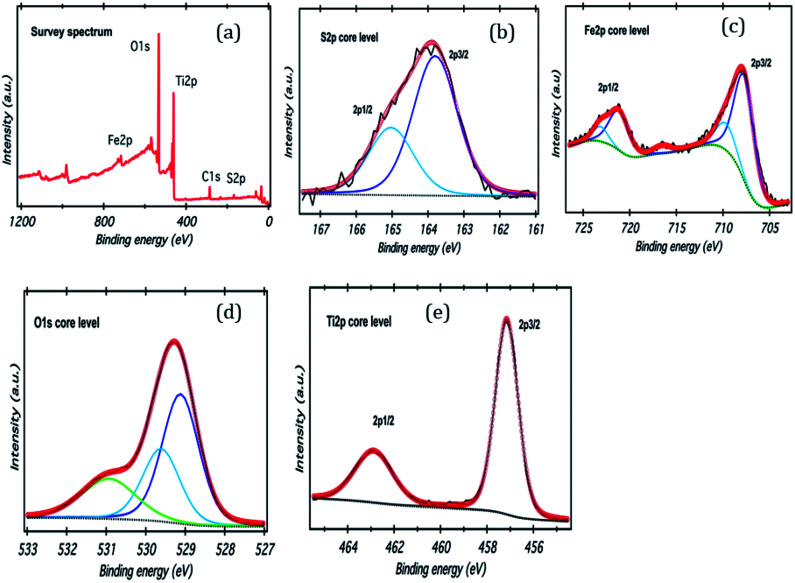
(a) XPS survey scan of FeS_2_/TiO_2_, (b–e) high resolution XPS spectra of S 2p, Fe 2p, O 1s, and Ti 2p.

Scanning electron microscope images of FeS_2_ nanocrystals and TiO_2_/FeS_2_ composite nanocrystals are presented in Fig. 2(a and b) respectively. Nanocrystals of FeS_2_ are uniformly spherical shapes while composite nanocrystals are mostly spherical with a few being elongated with increasing grain size. Micrographs confirmed that the grain size of FeS_2_ is smaller than the TiO_2_/FeS_2_ nanocomposite system. These observations correspond with XRD findings, as discussed in the above section. Compositional analysis was confirmed from energy dispersive X-ray spectroscopy (EDXS) measurements as demonstrated in Fig. 3. The spectra of a composite sample confirmed the presence of Ti, O, Fe, and S from constituents, while the Cu and C signal originated due to carbon tape and Cu grids. The weight percentage (wt%) of each element obtained from EDXS are presented in Table 1.

**Table 1:** Effect of catalyst dose, solution pH, and MB concentration on photocatalytic reaction rate

Catalyst dose^a^ (g L^−1^)	*k*_app_ (min^−1^)	*R* ^2^	Solution^b^ (pH)	*k*_app_ (min^−1^)	*R* ^2^	Initial concentration^c^ (mg L^−1^)	*k*_app_ (min^−1^)	*R* ^2^
0.25	0.0377	0.9793	2	0.0045	0.9216	15	0.0402	0.9981
0.5	0.0316	0.9876	5	0.0297	0.9444	25	0.0316	0.9834
1	0.0409	0.9346	7	0.0377	0.9793	50	0.0215	0.9974
			9	0.0408	0.9334	75	0.0017	0.9445

a MB = 25 mg L^−1^, pH = 5, light source = sunlight.

b MB = 25 mg L^−1^, catalyst = 1 g L^−1^, light source = sunlight.

c pH = 7, catalyst = 1 g L^−1^, light source = sunlight.

### 3.3 Chemical properties

XPS analysis of the TiO_2_/FeS_2_ nanocomposite is shown in Fig. 3. The wide scan spectra of TiO_2_/FeS_2_ (Fig. 3(a)) consists of the peaks for S, Ti, Fe, and O as major elements. High resolution spectra of S 2p (Fig. 3(b)) shows two peaks centered at 163.8 eV and 165.0 eV corresponding to the S 2p_3/2_ and S 2p_1/2_ of the sulfur binding energy in FeS_2_. A high intensity peak (Fig. 3(c)) appeared at 707.7 eV and is the characteristic of pyrite (Fe 2p_3/2_).^33,34^ The O 1s peak located at binding energies of 529.0 eV (due to oxygen bonding with Ti species), 529.7 eV (due to oxygen vacancies) and 531.0 eV (due to O_2_^−^, OH^−^, and H_2_O) are presented in Fig. 3(d), respectively. Doublet peaks appearing at binding energies of 458.1 and 463.9 eV belong to Ti 2p_3/2_ and Ti 2p_1/2_ in the TiO_2_ (Fig. 3(e))^35^ nano-composite system indicating the presence of Ti^4+^ species.

### 3.4 Optical spectroscopy

**Fig. 4:**
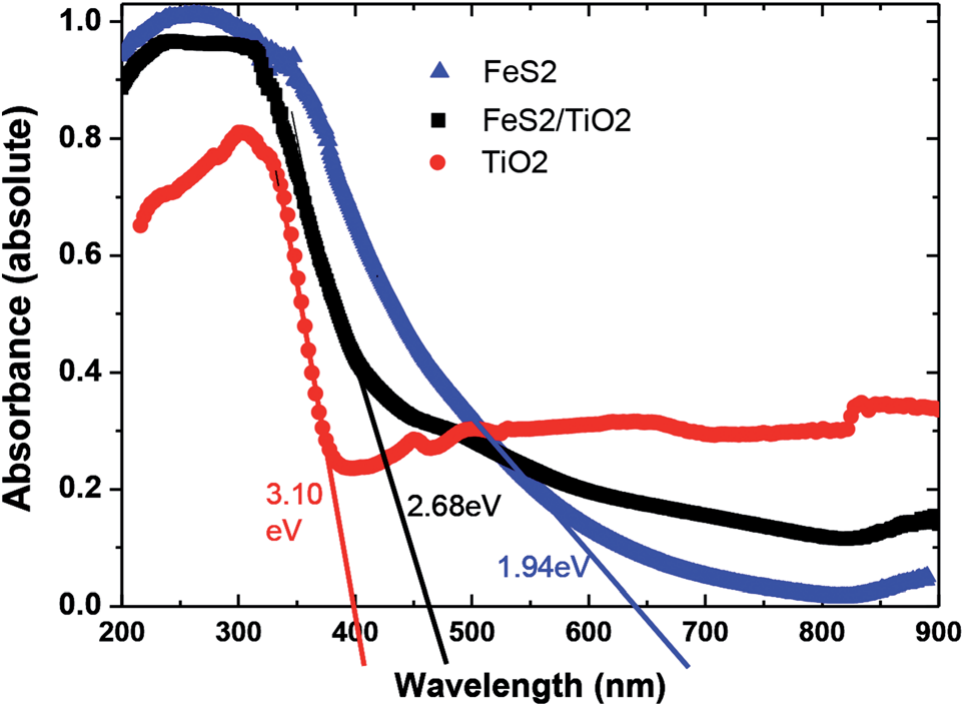
UV-Vis absorption spectra of nanocrystals TiO_2_, FeS_2_, and composite TiO_2_/FeS_2_.

UV-Vis absorption spectra of nanocrystals TiO_2_, FeS_2_, and composite TiO_2_/FeS_2_ are plotted in Fig. 4. The maximum absorbance of the TiO_2_ sample is 80% while the TiO_2_ and nanocomposite sample has maximum 100% absorbance at a lower wavelength range. The band gap energy of all samples was calculated using the following equation:

1 *E*_g_ = *hc*/*λ*_g_

where *λ*_g_ is the absorption onset wavelength (nm) of the exciting light, *c* is velocity of light and *h* is Plank's constant. The band gap energy values of TiO_2_ and FeS_2_ nanocrystals calculated using the above equation were found to be 3.10 eV and 1.94 eV, respectively. Interestingly, we observed that the TiO_2_/FeS_2_ nanocomposite sample band gap energy lies in a range around 2.68 eV. These results are very close to our prediction because the thrust of our study was to reduce the bandgap energy of a TiO_2_ un-doped system by combining with the FeS_2_ nanocrystals. Similarly, a polymer solar cell based on P_3_HT = FeS_2_NCs was reported^21^ and the results showed that a combination of polymer (P_3_HT) and FeS_2_ can contribute to an extended photovoltaic response in the red light region similar to literature^36,37^ reports that FeS_2_ is a narrow bandgap semiconductor (0.95–1.2 eV) showing absorbance across the entire visible region, and presence of sulfur in composite and pure FeS_2_ is indicated by a tail broadening of the peak.^25^

**Fig. 5:**
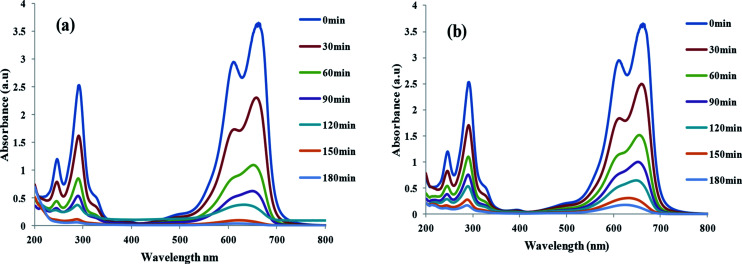
UV-Vis spectra of MB degradation in solutions containing TiO_2_/FeS_2_ in (a) sunlight and (b) visible light(dye concentration = 25 mg L^−1^, catalyst dose = 1 g L^−1^).

### 3.5 Photocatalytic degradation

The complete UV-Vis scanning spectra of a MB solution before (contact time zero) and after addition of TiO_2_/FeS_2_ catalyst recorded at regular intervals after direct sunlight and visible light (*λ* = 390–700 nm) exposure are presented in Fig. 5(a) and (b), respectively. From the UV-visible absorption scans rapid decrease in absorbance peaks can be noticed after addition of the nanocatalyst in both cases indicating high catalyst activity under varied irradiation sources. No new peaks were formed apart from two major peaks at 665 and 275 nm indicating lack of mineralization products and complete degradation of MB after 180 min of exposure. Absorption peaks in the UV region can be ascribed to the presence of aromatic rings such as benzene; naphthalene, *etc.* while the presence of chromophores containing a long conjugated π system are responsible for absorption in the visible region.^38,39^ Seemingly straight line spectra achieved after 180 min irradiation in the case of direct sunlight irradiation indicates increased photocatalytic efficiency in natural sunlight more than in visible irradiation attributed to the presence of UV in natural sunlight. Therefore, higher degradation was achieved in case of direct sunlight.^40^

**Fig. 6:**
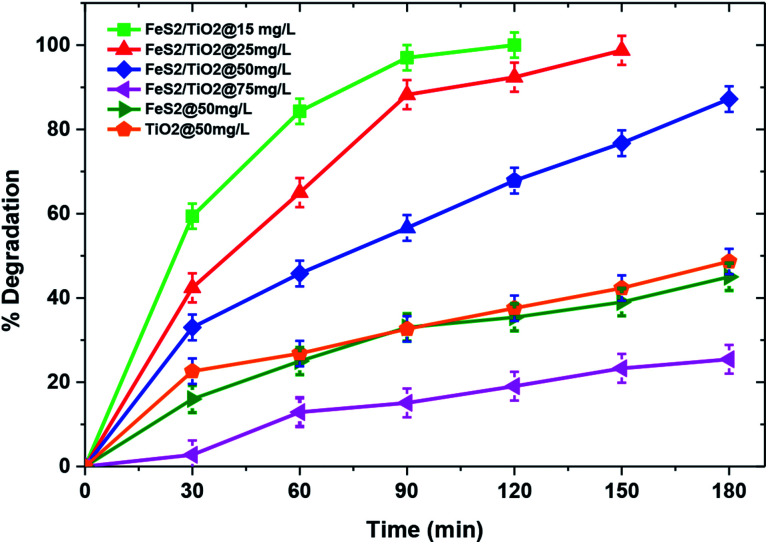
Effect of initial dye concentration and catalyst on photocatalytic degradation of MB(pH = 7, catalyst dose = 1 g L^−1^, light source = sunlight).

Comparison of photocatalytic activities of TiO_2_/FeS_2_, pristine TiO_2_, and FeS_2_ are illustrated in Fig. 6. This experiment was carried out to check catalyst activity of the TiO_2_/FeS_2_ nanocomposite and as synthesized TiO_2_ and FeS_2_ at pH = 7 and MB concentration of 50 mg L^−1^ showing degradation of MB is in a decreasing order *i.e.*, TiO_2_/FeS_2_ (97.2%) > TiO_2_ (48.64%) > FeS_2_ (45%). These results indicate that TiO_2_/FeS_2_ has degraded nearly double the amount of MB in 180 min as compared to the individual constituents. This is because FeS_2_ has a band gap of 1.00 eV; thus, it can be easily excited using visible light. However, decreased photoactivity of pure FeS_2_ rather than the nanocomposite was due to an elevated electron–hole recombination rate.^41^ The higher MB degradation by TiO_2_/FeS_2_ compared to TiO_2_ and FeS_2_ alone was due to efficient electron–hole separation,^28^ resulting in an increased number of charges participating in the photocatalytic degradation of MB. Another possible reason for enhanced photocatalytic degradation might be an increased oxidation–reduction potential of the composite *i.e.*, TiO_2_/FeS_2_.^25^ The above results confirmed that TiO_2_/FeS_2_ exhibited enhanced degradation of MB compared to pristine TiO_2_ or FeS_2_; therefore, remaining experiments were conducted using the TiO_2_/FeS_2_ nanocomposite under solar light irradiation.

### 3.6 Effect of initial dye concentration

The initial pollutant concentration plays a very important role in photocatalytic reactions from both application and mechanistic points of view. Fig. 6 illustrates that complete degradation was achieved for an initial pollutant concentration of 25 mg L^−1^; however, there was significant decline in the MB degradation to 25.3% with an increase in initial dye concentration to 75 mg L^−1^ within 180 min of irradiation. This decline in catalytic activity with increasing pollutant concentration in the solution may be attributed to lower transparency of the solution to light with increasing pollutant molecules and a longer path length for light to reach the catalyst's surface which, in turn, triggers lesser photocatalytic reactions.^42^

### 3.7 Effect of solution pH

**Fig. 7:**
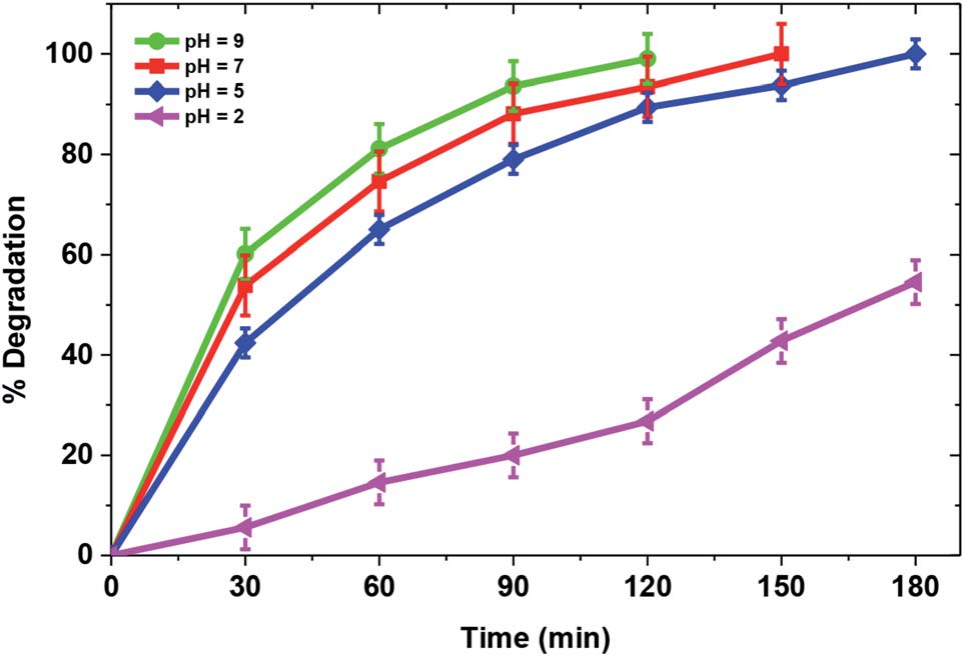
Effect of pH on photocatalytic degradation of MB(MB = 25 mg L^−1^, catalyst = 1 g L^−1^, light source = sunlight).

Solution pH is an important parameter that can affect efficiency of the photocatalytic process. Fig. 7 demonstrates the effect of solution pH on degradation of MB over a TiO_2_/FeS_2_ nanocomposite. Drastic changes in catalytic activity were observed with solution pH change from normal solution pH of 5 to acidic pH 2 with corresponding degradation of 25 mg L^−1^ MB from 42.8% at pH 2 to 100% at pH 5 within 180 min of irradiation. Further increases in pH from 5 to 7 and 9 achieved complete degradation at 150 and 120 min, respectively showing increased catalytic activity in alkaline conditions. These results could be explained with the help of point of zero charge of TiO_2_/FeS_2_ (pH_zpc_ = 3.8) determined by the salt addition method using 0.1 M KCl.^43^ Consequently, the surface of the composite is positively charged at pH lower than 3.8 and electrostatic repulsion between the cationic dye molecule and positively charged catalyst surface in acidic pH would result in lower adsorption of the MB dye over the catalyst's surface resulting in lower degradation efficiency. On the contrary, at pH higher than the pH_zpc_ the electrostatic attraction between the dye molecules and negatively charged surface of TiO_2_/FeS_2_ results in higher photocatalytic degradation at pH 5. Also, basic conditions favor production of hydroxyl radicals^43^ which would further increase the catalytic degradation of MB.

### 3.8 Effect of catalyst dose

**Fig. 8:**
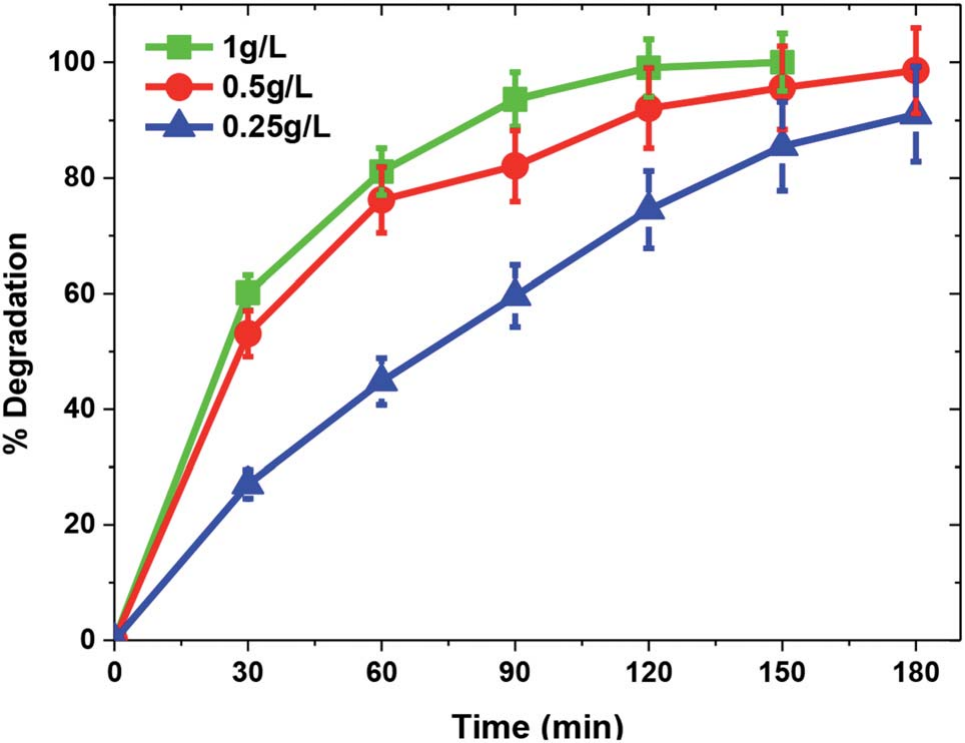
Effect of catalyst loading on photocatalytic degradation of MB(MB = 25 mg L^−1^, pH = 5, light source = sunlight).

Another important factor influencing photocatalytic degradation rate is catalyst dose. A set of experiments was carried out to find the optimum catalyst concentration by increasing the TiO_2_/FeS_2_ nanocomposite dose from 0.25–1 g L^−1^ at normal solution pH = 5 and MB concentration of 25 mg L^−1^. A characteristic behavior observed in heterogeneous photocatalysis is that with an increase in catalyst dose, the degradation percentage is also increased.^44^Fig. 8 illustrates a similar pattern for the photocatalytic degradation of MB with increments in catalyst dose from 0.25–1 g L^−1^ having a maximum of 100% degradation for 25 mg L^−1^ MB at 1 g L^−1^ catalyst dose within 120 min while 98.56% and 74.04% degradation efficiency was achieved at 120 min solar irradiation over 0.5 g L^−1^ and 0.25 g L^−1^ of TiO_2_/FeS_2_, respectively. Increased catalyst dose refers to an increased number of catalyst active sites per unit volume with more available surface area, which leads to the production of more OH^−^ radicals and higher degradation rates.^45,46^

### 3.9 Reusability

**Fig. 9:**
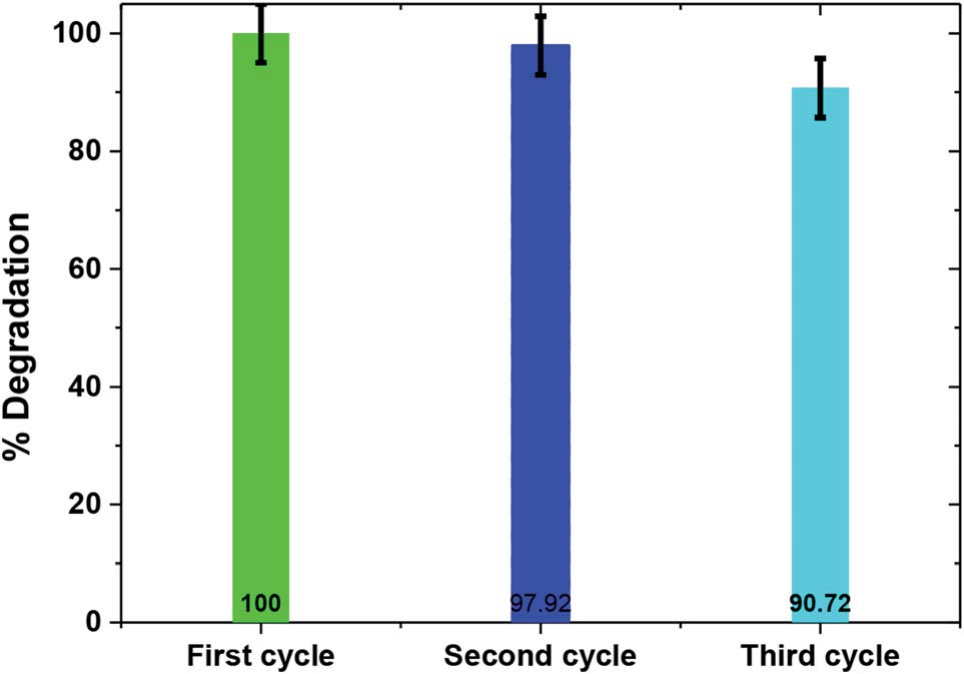
Photocatalytic efficiency of TiO_2_/FeS_2_ upon recycled application (MB = 25 mg L^−1^, pH = 5, light source = sunlight).

Catalyst stability is a key factor for large scale applicability of any photocatalyst. To determine the photocatalyst's stability, the TiO_2_/FeS_2_ nanocomposite was recovered and used multiple times to degrade fresh MB solutions. After each reuse experiment the catalyst was simply filtered, dried in an oven at 80 °C, and reused without any further modification. Fig. 9 depicts that the TiO_2_/FeS_2_ nanocomposite upon reuse retained high catalytic activity (100% to 97.2% and 90.72% by fresh, 2^nd^ use, and 3^rd^ use, respectively) with only about 9% loss in photocatalytic efficiency after the third cycle. This slight decrease in the rate of MB degradation upon reuse may be due to accumulation of organic intermediates, as detected by GCMS on the surface of a catalyst sample, thus affecting the adsorption of pollutant and, in turn, decreasing degradation.^47,48^ Apart from catalyst agglomeration, photo-dissolution, oxidative decomposition, and photo-corrosion, there are different processes in a photocatalytic reaction that negatively affect the surface of the catalyst, thus reducing its reusability and stability.^49^

### 3.10 GCMS analysis

**Fig. 10:**
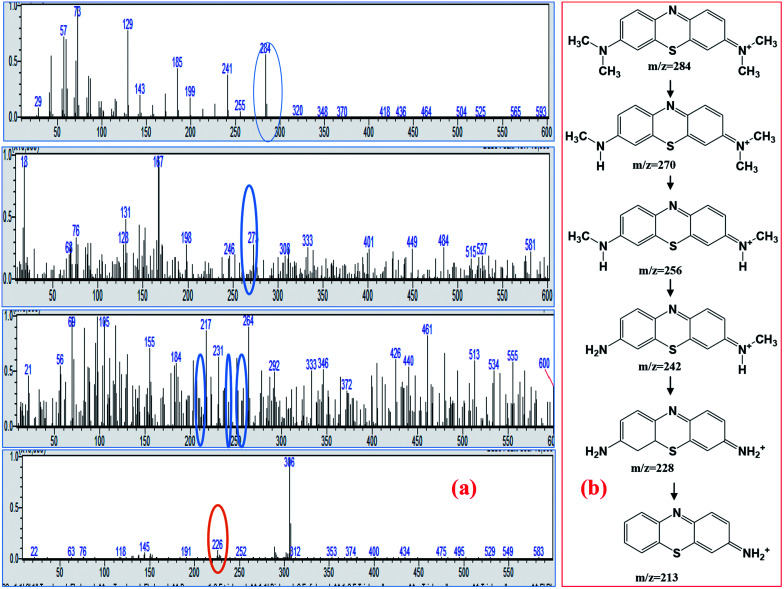
(a) Mass spectra of MB solution treated with TiO_2_/FeS_2_, (b) possible degradation mechanism of MB based on MS spectra.

For detailed insight into the degradation mechanism of methylene blue by the TiO_2_/FeS_2_ nanocomposite, GCMS analysis was carried out on selective samples under optimized photocatalytic conditions. Based on mass spectra of the intermediate products formed before complete mineralization, it can be proposed that the MB degradation reaction occurs *via* high-energy electron impact. The MB degradation mechanism is proposed, and their respective mass spectra are shown in Fig. 10(a) and (b). High energy electrons generated as TiO_2_/FeS_2_ when irradiated with solar light are able to dissociate the chemical bonds in MB. By far the most susceptible point for electron dissociation is the bond between H_3_C–N atoms having a bond energy of 3.07 eV.^50^ The results appear in a successive demethylation reaction pathway leading to MB degradation. Due to degradation of MB, presence of demethylated molecules such as Azure A, Azure B and C, and thionine having mass to charge ratios of 270, 256, and 228, respectively are reported in literature.^51,52^ In the degradation of MB, the S–Cl bond broke first due to low bond disassociation energy, leading to the detection of Cl^−^ at the beginning of the reaction. The structure of the *m*/*z* = 284 ion corresponds to the MB cation which is due to [MB + H]^+^. Due to bonds broken between S⋯Cl, C⋯SO_3_H, and C⋯NH_2_ during degradation of the MB process Cl^−^, SO_4_^2−^, and NO_3_^−^ were detected.^53^ Phenyl thiophene present in the form of protonated phenyl thiophene having *m*/*z* = 138 was also detected in this study.^54^

**Fig. 11:**
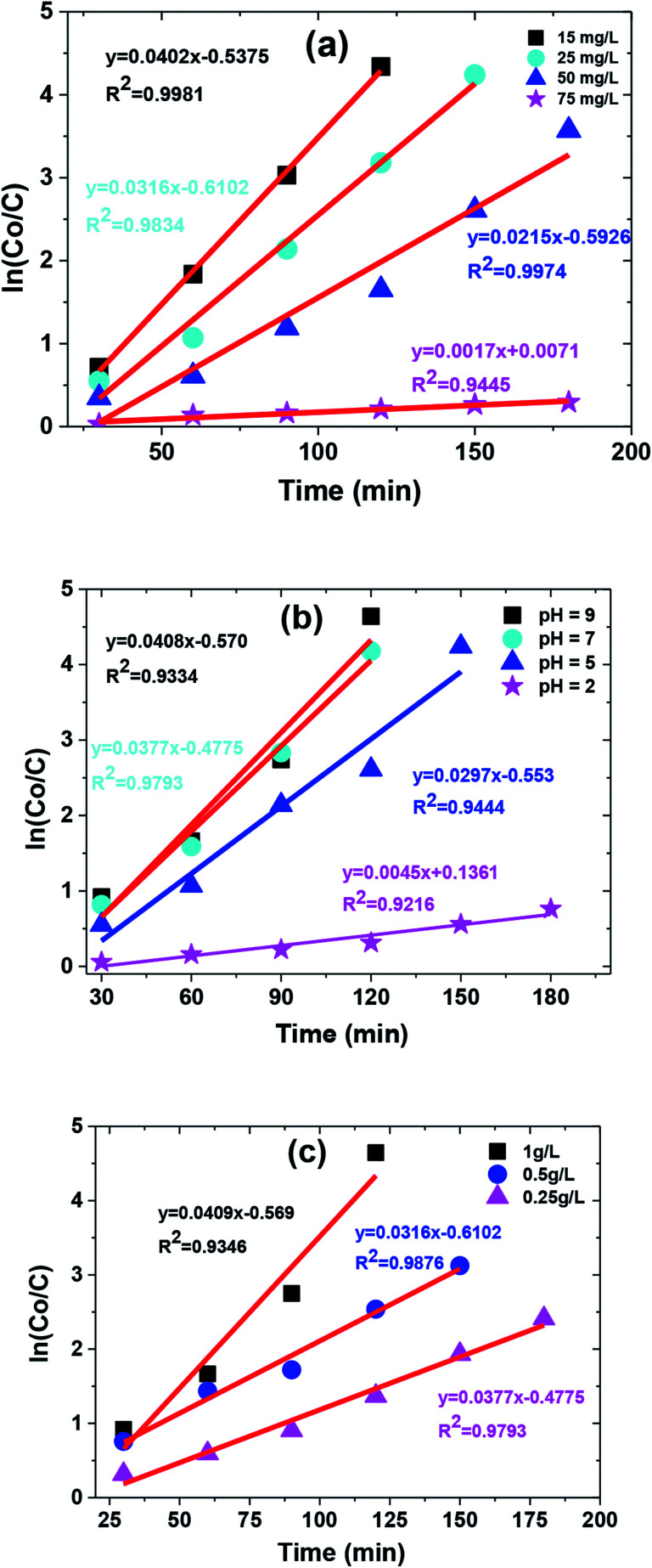
The kinetic curve of degradation catalyzed by TiO_2_/FeS_2_ under (a) different MB concentration, (b) different pH conditions, and (c) catalyst dose.

Langmuir–Hinshelwood kinetics generally fit well with photocatalytic degradation reactions at the solid–liquid interface.^55^ Kinetic parameters involved in MB degradation were evaluated by fitting this equation to the experimental data. Basic L–H relationship is given in eqn (2).2
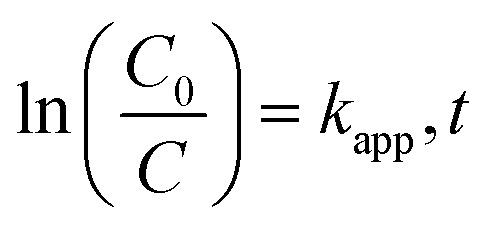
where ‘*C*_0_’ represents initial dye concentration, ‘*C*’ represents the concentration in solution of MB at time ‘*t*’ (min), and *k*_app_ is the rate constant (min^−1^). Increase in MB concentration decreased rate constant from 0.0402 to 0.0017 (min^−1^) as shown in Table 1. A similar trend was observed in the case of pH with a maximum rate constant value obtained at pH 9 (*i.e.*, 0.0408 (min^−1^)). However, the rate constant increased with increase in catalyst dose as shown in Table 1. The kinetic plot between ‘ln(*C*_0_/*C*)’ and time ‘*t*’ for operational parameters (*i.e.*, increasing dye concentration, solution pH, and catalyst dose) are shown in Fig. 11(a)–(c).

### 3.11 Reaction mechanism

Three stages on which complete photocatalytic process is generally reliant are light harvesting, separation of photogenerated charges, and interfacial reactions.^50^ For an efficient photocatalyst, implementation at each stage is necessary. Here we present the reaction mechanism of TiO_2_/FeS_2_ nanocomposite which can efficiently separate photogenerated charge carriers (h^+^ & e^−^) due to formation of a heterojunction between TiO_2_ and FeS_2_ and satisfied energy level position of TiO_2_/FeS_2_ composite *versus* vacuum energy.^28^ FeS_2_ with narrower band gap is easily excited by visible and sunlight, which initiates the generation of photoelectrons and holes. Photoelectrons in the conduction band of FeS_2_ can be transferred to CB of TiO_2_, leaving holes in VB of FeS_2_; as a result, the recombination rate of charge carriers was decreased successfully, and the photocatalytic activity was improved. Meanwhile, the generated VB hole can react with water to form H^+^ and an OH˙ radical and then superoxide radical anions are generated by reaction of CB electrons with dissolved oxygen molecules, (O_2_˙) which undergo a series of reactions forming OH˙, ultimately reacting with target pollutant (MB) and forming nonhazardous end product as depicted in eqn (3) through (11).

3 FeS_2_ + *hν* = FeS_2_ (h^+^ + e^−^)

4 TiO_2_/FeS_2_ (h^+^ + e^−^) = FeS_2_ (h^+^) + TiO_2_ (e^−^)

5 FeS_2_ (h^+^) + MB = CO_2_ + H_2_O (oxidation product)

6 TiO_2_ (e^−^) + MB = CO_2_ + H_2_O (reduction product)

7 O_2_ + TiO_2_ (e^−^) = O_2_˙^−^

8 O_2_^−^ + FeS_2_ (h^+^) = HO_2_˙

9 TiO_2_ (e^−^) + HO_2_˙ = H_2_O_2_

10 H_2_O_2_ + TiO_2_ (e^−^) = OH˙ + OH

11 OH˙ + MB = CO_2_ + H_2_O (degradation product)

## 4. Conclusions

The aim of this study was to synthesize a solar/visible light active nanocomposite for the degradation of a commonly used hazardous textile dye *i.e.*, MB. A TiO_2_/FeS_2_ nanocomposite was synthesized successfully and exhibited good crystallinity. FeS_2_ nanoparticles were uniformly dispersed on TiO_2_. The band gap of TiO_2_ exhibited a red shift after addition of FeS_2_. The obtained TiO_2_/FeS_2_ nanocomposite showed excellent catalytic activity in degradation of MB. Furthermore, a repeatability test showed that TiO_2_/FeS_2_ had good stability and reusability. In comparison, TiO_2_/FeS_2_ showed a higher catalytic activity than FeS_2_, thereby proving the advantage of FeS_2_ addition in the composite showing the highest degradation of MB under both direct sunlight and visible light *i.e.*, 100% and 97.2%, respectively in the case of TiO_2_/FeS_2_. The nanocomposite TiO_2_/FeS_2_ showed increased degradation of the organic pollutant; which was confirmed by the increased rate of chemical reaction following pseudo first-order reaction kinetics with the highest rate constant value of 0.0408 m^−1^ and having *R*^2^ 0.9981.

## Conflicts of interest

There are no conflicts to declare.

## Supplementary Material
